# Supplementary material for: Global hypo-methylation in a proportion of glioblastoma enriched for an astrocytic signature is associated with increased invasion and altered immune landscape
Source: eLife. 2022 Nov 22;11:e77335. doi: 10.7554/eLife.77335 (PMC9681209; doi:10.7554/eLife.77335)
Supplement: Figure 2—source data 1. [file elife-77335-fig2-data1.zip › Figure_2_source_data_1/Figure_2A/homerResults/motif11.similar.html]

motif11

## Information for motif11

A
C
T
G
T
G
C
A
T
G
A
C
T
G
C
A
T
G
A
C
T
A
G
C
G
T
A
C
G
C
T
A
A
C
T
G
G
A
T
C
G
T
A
C
G
T
A
C
G
T
A
C
A
G
T
C
G
T
A
C
T
G
A
C
T
G
A
C
A
T
G
C
G
T
A
C
G
C
T
A
T
A
C
G
A
C
G
T
T
G
C
A
T
G
A
C
T
A
C
G
A
T
G
C
G
A
T
C
T
C
G
A
T
A
C
G
T
A
G
C
G
T
A
C
G
A
C
T
A
G
T
C
G
A
C
T
T
C
G
A
  
Reverse Opposite:  

A
G
C
T
C
T
G
A
A
C
T
G
C
T
G
A
C
A
T
G
A
T
C
G
A
T
G
C
A
G
C
T
C
T
A
G
T
A
C
G
A
T
G
C
A
C
T
G
A
C
G
T
T
G
C
A
A
T
G
C
C
G
A
T
C
A
T
G
A
T
C
G
A
C
T
G
A
C
T
G
C
A
T
G
T
C
A
G
C
A
T
G
C
A
T
G
C
A
T
G
C
T
A
G
T
G
A
C
C
G
A
T
A
C
T
G
A
T
C
G
A
C
T
G
A
C
G
T
A
C
T
G
A
C
G
T
T
G
A
C
  

|  |  |
| --- | --- |
| p-value: | 1e-23 |
| log p-value: | -5.336e+01 |
| Information Content per bp: | 1.507 |
| Number of Target Sequences with motif | 25.0 |
| Percentage of Target Sequences with motif | 1.08% |
| Number of Background Sequences with motif | 2.2 |
| Percentage of Background Sequences with motif | 0.06% |
| Average Position of motif in Targets | 94.7 +/- 47.8bp |
| Average Position of motif in Background | 115.1 +/- 55.8bp |
| Strand Bias (log2 ratio + to - strand density) | 0.9 |
| Multiplicity (# of sites on avg that occur together) | 1.24 |
| Motif File: | file (matrix) reverse opposite |

### Similar de novo motifs found

|  |  |  |  |  |  |  |  |
| --- | --- | --- | --- | --- | --- | --- | --- |
| Rank | Match Score | Redundant Motif | P-value | log P-value | % of Targets | % of Background | Motif file |
| 1 | 0.664 | T C A G A C G T T G C A G C T A C A T G A G T C A T G C T C G A C A T G A G T C G T A C G T C A T A G C G A T C G T C A T G A C A T C G A G T C G A T C C G T A T C A G A C T G G T C A T G C A T C A G G T A C G T A C C T G A A T C G A G C T T G A C G T C A T A G C A G C T T C G A G T A C T C G A A T G C G A T C C G T A T C G A C T G A T C G A G T A C T C A G A T G C T G A C C T G A C A T G A G T C | 1e-13 | -30.686188 | 0.73% | 0.07% | motif file (matrix) |
| 2 | 0.675 | T G A C A G C T G T C A G C A T C T G A A G T C G T A C G C T A T C A G A C G T T G C A C G T A A T C G G T A C T A G C C G T A A T C G G T A C G T A C G T C A T G A C A C G T G T C A G A T C C T G A T G A C T G A C C G T A A T C G T A G C G C T A C T G A T C A G T A G C G A T C G T C A T A C G A G T C T G A C G C T A A G T C G A C T C G T A G A T C C G T A | 1e-12 | -29.072671 | 0.56% | 0.03% | motif file (matrix) |
| 3 | 0.627 | G A T C A T G C C G T A T C A G G T A C A G T C G T C A A G T C A G C T T C G A A G T C G T C A A G T C G A T C C G T A C T A G A C G T G T A C C G T A C T A G | 1e-7 | -18.171152 | 0.69% | 0.11% | motif file (matrix) |
